# Mekeres’ Psychosocial Internalization Scale: A Scale for the Evaluation of Aesthetic Prejudice in Victims of Accidents and Violence

**DOI:** 10.3390/healthcare9111440

**Published:** 2021-10-26

**Authors:** Florica Voiță-Mekeres, Camelia Liana Buhaș, Gabriel Mihai Mekeres, Cristina Tudoran, Mariana Racovita, Cosmin Ioan Faur, Mariana Tudoran, Ahmed Abu-Awwad, Nuțu Cristian Voiță, Teodor Andrei Maghiar

**Affiliations:** 1 Faculty of Medicine and Pharmacy, University of Oradea, 410087 Oradea, Romania; mekeres_florina@yahoo.com (F.V.-M.); cameliabuhas@uoradea.ro (C.L.B.); racovita.mariana@student.uoradea.ro (M.R.); voita.nutucristian@student.uoradea.ro (N.C.V.); teodormaghiar@yahoo.com (T.A.M.); 2 Department VII, Internal Medicine II, Discipline of Cardiology, University of Medicine and Pharmacy “Victor Babes” Timisoara, 300041 Timisoara, Romania; tudoran.mariana@umft.ro; 3 Center of Molecular Research in Nephrology and Vascular Disease, Faculty of Medicine, University of Medicine and Pharmacy “Victor Babes” Timisoara, 300041 Timisoara, Romania; 4 County Emergency Hospital, 300736 Timisoara, Romania; 5 Department XV, Orthopedy-Traumatology, Urology and Imagistical Medicine, Discipline of Orthopedy, University of Medicine and Pharmacy “Victor Babes” Timisoara, 300041 Timisoara, Romania; faur.cosmin@umft.ro (C.I.F.); ahm.abuawwad@umft.ro (A.A.-A.)

**Keywords:** aesthetic prejudice, aesthetic evaluation methods, Mekereș Psychosocial Internalization Scale, psycho-social impact

## Abstract

Background and objectives: One important forensic activity is the assessment of aesthetic injuries where expert criteria and analysis are insufficiently outlined due to the subjective elements related to the traumatized victim. Unaesthetic morphological changes may occur due to various circumstances committed under the Penal Code, resulting in permanent unaesthetic morphological scarring. Considering that most of the existing scales for the assessment of aesthetic prejudices refer only to morphometric changes, our aim was to create a modern method for the evaluation of aesthetic damage that also considers its social and psychological consequences. Materials and Methods: In this study, we developed the Mekereș Psychosocial Internalization Scale (MPIS), which proposes a clear boundary between the presence or absence of aesthetic damage. The traumatized person is evaluated after a minimum of six months (in the case of an average scar, necessary for defining the character of the scar) to assess changes in the physiognomy or even alterations in the victim’s aesthetic perception of their own body. Our study was conducted on 103 patients with scars, and the results were compared to 101 controls (subjects without scars). Results: Individuals with scars have a distorted perception (compared to controls) of the support provided by significant people [t (202) = 2.473; *p* = 0.01]. Hypothetically, they will most likely exhibit a nuanced socio-cognitive and psycho-emotional vulnerability that may be the source of future dysfunctions. The fidelity of the MPIS scale was estimated by employing Cronbach’s alpha coefficient, resulting in a value of 0.934 (15 items). The exploratory factorial analysis with Varimax rotation mode sustains a single dominant factor, indicating a good internal consistency. The results of this study provide evidence regarding the psychosocial or psychometric worthiness of MPIS. Conclusions: MPIS can be used for research and as an instrument to assess aesthetic damage or disfigurement by forensic physicians and lawyers.

## 1. Introduction

Due to modern-day evolution and gender equality policies, physical appearance also tends to increase in importance in today’s society. For some highly paid professions that do not require a high degree of education such as modeling, advertising, television career, and social networking, physical appearance represents almost the sole criterion for employment [1,2]. The negative consequences of aesthetic damage are perceived mainly in the family and at the professional level in addition to the suffering induced by self-awareness of the aesthetic wounds or scars [3,4,5].

This study originates from the hypothesis that bodily harm may be objective, but for the elements of moral suffering induced by the presence of scars, the objective criteria are missing [6]. Several attempts to quantify the amplitude and impact of aesthetic damage on professional development, as well as on the psycho-social well-being of these patients, have been proposed [7,8,9]. Hodin and Greff’s aesthetimetric method quantifies aesthetic damage according to the morphological characteristics of the scar [10]. This method is difficult to put into practice because it divides the face, the previous plane into 72 geometric figures, in which two lateral facial planes of 25 areas are added together, resulting in 122 sectors. Each lesion may be of interest to one or more sectors. To determine the degree of disfigurement, each sector is scored according to the basic coefficient, the coefficient of correction of the fracture, the coefficient of difference concerning the tegumentary plane, the plastic coefficient, the color coefficient, and the texture. Afterward, the points per sector are gathered, and then a calculation for all the sectors affected is made [11,12,13].

The Patient and Observer Scar Assessment Scale (POSAS) was proposed by Draaijers et al. [14] and allows a structured clinical evaluation of the quality of the scars, reflecting both the observer’s and the patient’s opinions in the evaluation of the scars. This tool is consistent and reliable in the case of burn scars. The characteristics of the scars such as vascularization, pigmentation, firmness, foldability, the size of the affected area, and length of the scar are scored [10,15]. The Vancouver Scar Scale (VSS), also called the Burn Scar Index, first described by Sullivan, is a method for measuring scars and is probably the most recognized method for the assessment of burn scars. This scale evaluates the risk factors for the development of hypertrophic scars and the effectiveness of therapy. This scale analyzes the vascularization, pigmentation, foldability, and height of the scars to diagnose the hypertrophic ones [11,14,16].

The objectives of this research are to elaborate a method for the assessment of aesthetic prejudice by creating a scale with a clear delineation between the presence or absence of aesthetic damage. We propose a method to quantify aesthetic damage entitled “The Mekereș Psychosocial Internalization Scale” (MPIS) based on major criteria, scored with three points, and minor criteria, scored with one point. In this scale, the awareness of the presence of scars, the gender of the victims, the morphological characteristics of the scar, the negative impact on the social interaction affecting the professional development of the individual, and the presence of post-traumatic disorders are taken into account [17,18].

In the first step of the development and validation process of the MPIS scale, we defined the conceptual domain of the construct by investigating and analyzing numerous data from the specialized literature. Content validity was obtained through the critical analysis of the concept’s operationalization and the examination of the variables’ internal consistency. After pilot testing and operating the latest changes, the MPIS was prepared to be analyzed from the perspective of construct validity. Convergent validity highlighted that the measures of the concept are correlated. The procedure for estimating the convergent validity, for example, the examination of the saturations (factor loading) of the variables in the elements extracted by the exploratory factorial analysis (EFA), is a more appropriate method in this context. The predictive validity will be established subsequently, during the study.

We started from the following hypotheses: 1. We assumed that MPIS will have a significant discriminatory capacity depending on the categories of investigated patients; 2. We presumed that the MPIS scale will have good fidelity and validity.

Improvement of current methods for the assessment of aesthetic prejudice by removing the subjectivism of the forensic physician represents an essential aspiration for physicians and especially for patients [15,19]. By replacing the current aesthetic method, which is about 50 years old, with a modern one that takes into account the social and psychological dimension of the aesthetic damage, we are trying to offer a practical tool for the establishment of compensations from insurance companies regarding the aesthetic damage [19].

## 2. Materials and Methods

### 2.1. Study Population

The study includes a total number of 204 participants aged between 18 and 81 years (mean age of 46.68 ± 19.03 years), 105 of which are women (51.5%) and 99 (48.5%) are men.

They were selected on a randomized basis from all patients who attended the forensic medicine and plastic surgery services of the County Hospitals from two large cities in Romania (Cluj and Oradea). Of all evaluated patients, 252 were considered eligible. They were briefly informed about the purpose of the study and were asked to participate after signing an informed consent. 224 agreed to sign the informed consent form and were included in the study, but 20 subjects did not meet the inclusion criteria and were excluded, resulting in a study population of 204 patients.

Inclusion criteria were:

1. subjects aged over 18 years;
2. patients who suffered a traumatic injury (as a result of violence/aggression or accident) at the level of the face and/or neck (visible), resulting in post-traumatic scarring, at least 6 months ago (to properly assess the stage of healing) and who received surgical or conservative treatment;
3. patients without a pre-existing pathology of the face;
4. the willingness and ability to sign an informed consent form.

Exclusion criteria:

1. subjects under 18 years or not able to read/understand/sign the informed consent;
2. refusal to participate in the study or to sign the informed consent form;
3. patients with severe acute mental illness and/or inability to understand questionnaires related to psychosocial internalization and perceived social support;
4. patients with pre-existing pathology of the face;
5. other etiology of the aesthetic damage (non-traumatic: surgery, oncology);
6. inability to understand the risks/benefits/expected results/lifestyle changes associated with cosmetic and restorative surgery.

The participants included in this study were divided into two homogeneous groups according to the presence/absence of scars: group A—consists of 103 patients with scars on the face and/or neck, and group B—represents the control group, which includes 101 individuals without scars.

The patients included in group A were treated, some by conservative measures, others by surgery, to remedy the aesthetic damage created by a traumatic lesion. Specifically, we focused on patients who had scarring on the face and the anterior cervical region.

### 2.2. Psychosocial Assessment of the Aesthetic Prejudice

We propose the term “psychosocial internalization” of the scar, which we defined as the limit to which a person can adapt himself to scarring from a social, familial, and psychological point of view. The minor scars, acquired in childhood, no longer affect a person in adulthood once the formation of the personality is complete and is considered part of the normal appearance of a person. Therefore, this no longer affects self-image, confidence, and relationships with others, and the patient no longer attempts to hide the scar [20]. Initial instructions underline the fact that people have different perspectives; therefore by employing MPIS, interested doctors can quantify the influence of the scar on a person’s mental status and feelings by using a selection of 15 listed questions. Patients with scars are asked to score from 1 to 5, each item on the MPIS. They were instructed to select a number that corresponds to their personal perspective. If the person is not sure, they are advised to circle the nearest number that would correspond to his or her perspective. In this study, we employed the initial version of the MPIS, developed in Romanian, and analyzed the answers provided by our patients who read and completed the items of the scale in this language. For further use and future research, a specialist psychiatrist who has proficient English skills translated the MPIS items into English and we used the forward-backward method to certify its accuracy.

### 2.3. Quoted Answers

The answers are presented as numbers from 1 to 5, where 1 represents “I don’t agree” and 5, “I totally agree”. The MPIS results are achieved by summing the total score, comprised between 15 and 75 points.

Interpretation of the MPIS: Based on the data collected from our patients, after debating with the psychiatrists and psychologists who examined the patients, and after a statistical analysis of the answers, we propose the following scoring: scores below 35 points signify that the affected person has managed to adapt themselves to the presence of the scar without having a psychosocial impact on their daily life, the threshold for cosmetic damage is ≥35 points, and in cases of scores exceeding 55 points after completing all plastic surgery and reparatory treatments, we propose the reintroduction of the term of disfigurement.

Interpretation of MPIS score:

- <35 points = psychosocial internalization of the scar;
- Between 35–54 points = aesthetic damage;
- ≥55 points = disfigurement.

The study was approved by the Ethics Committee of the Clinical Emergency Hospital Oradea Nr.20232/21.08.2017, by the Institute of Forensic Medicine Cluj-Napoca 5623/X/556/29.09.2017, and by the Service of Forensic Medicine of County Bihor 4530/X/285, and all patients signed a written informed consent.

### 2.4. Statistical Methods

A preliminary analysis was conducted, and since the Kolmogorov–Smirnov test showed a Gaussian distribution, we continued with parametric tests. Mean values and standard deviation (SD) were calculated for the two groups. *p* values under 0.05 were considered statistically significant.

Patients’ characteristics were analyzed by using the Chi-square test or Freeman–Halton extension of Fisher’s exact test. A preliminary examination of the correlational matrix showed that all MPIS items are positively correlated and the sphericity Bartlett test is statistically significant. The fidelity of the MPIS was estimated using the Cronbach alpha coefficient. Sample adequacy testing was implemented using the KMO (Kaiser–Meyer–Olkin) method, and the obtained values indicated that our sample met the basic conditions for the use of exploratory factorial analysis (EFA). EFA is a method that we consider effective for the detection of a structure of some common factors. We also used the factorial analysis method with Varimax rotation mode, which allows factors to correlate at various intensities. The Statistical Package for the Social Sciences v.20 (SPSS, Chicago, IL, USA) was employed to perform data analysis. The confirmation of the dominant factor in MPIS through EFA, as well as concurrent validation of the scale using statistical methods (SPSS), will support the utility of the tool in the areas presented above.

## 3. Results

The study group consisted of 103 patients with scars, 51 (49.5%) women and 52 (50.5%) men with a mean age of 46.68 ± 19.03 years. There were no statistically significant differences between the genders. Fifty-three subjects (51.5%) came from rural areas and 50 (48.5%) from urban zones. Additional information over demographic variables such as ethnicity, marital status at the time of our research, as well as profession and/or employment are presented in Table 1. From an educational point of view, 31 patients with scars (30.1%) declared themselves graduates of professional or secondary schools, 40 subjects were high school graduates (38.8%), and the other 32 participants (31.01%) graduated from university. From an occupational perspective, the experimental group included 42 (40.8%) patients with a low economic status, 38 (36.9%) with average income, and 23 patients (22.3%) with a high economic status.

### 3.1. Validation Study as a Tool Scale—MPIS—Objectives

1. Validation and establishing norms for the population;
2. Empirical examination of the relationship between MPIS and other relevant scales.

Our study comprised a sample of 103 participants with scars, residing in both rural and urban areas, and was heterogeneous in terms of their educational and economic level. Additional data regarding the experimental group are presented in the section allocated to the research participants.

We present the MPIS as well as the theoretical data that led to its development. Regarding the research objectives, we aim to validate the scale and establish norms for the population.

In the first phase of the research, we focused our attention on the starting statistical indices; therefore, in Table 2, we present the mean and standard deviations for each item and the total score of the MPIS.

### 3.2. Factor Structure of the MPIS

We believe that the MPIS represents a single methodology that covers the variation of results recorded in patients with scars (*n* = 103). A preliminary examination of the correlation matrix showed that all MPIS items are positively intercorrelated.

The Barlett sphericity test is statistically significant χ^2^ (*n* = 103) = 2002,302, *p* < 0.001, which supports the use of exploratory factorial analysis (Table 3). Sample adequacy testing was implemented using the KMO (Kaiser–Meyer–Olkin) method, yielding a value of 0.846, which means that the sample met the basic conditions for using exploratory factorial analysis. For a solid degree of adequacy of the sample for each variable, we used the “anti-image,” option which has diagonal values greater than 0.90 for eight items and values greater than 0.80 for seven items. These values indicate a good sample fit for each variable (Table 2).

Starting from studies available in the specialty literature, we demonstrated the facts that support, from an experimental point of view, the initial hypothesis.

**Figure 1:**
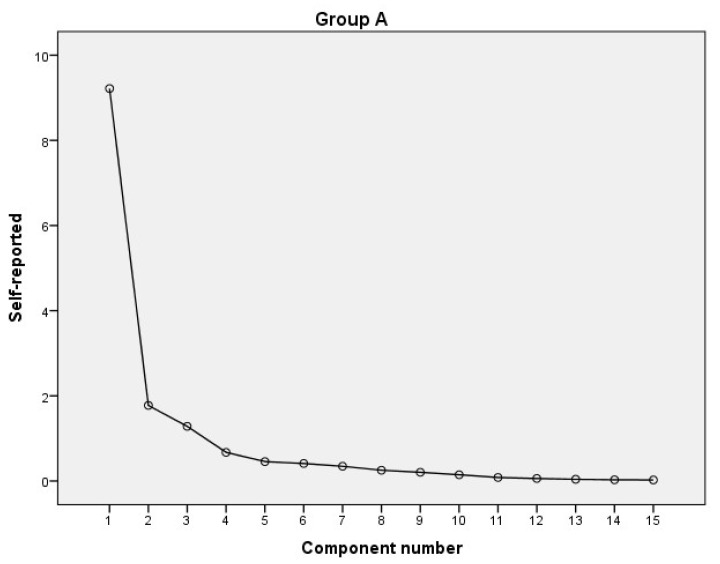
Graphical representation of the intensity of self-reported values for MPIS.

In Figure 1, we present the graphical representation of each factor of the possible 15 depicted on the abscissa and on the ordinate the self-reported values. As a result, we determined the factors that can lead to a suitable solution in the case of the MPIS. Thus, a single factor that represents 61.451 of the variance of the data was identified (Table 4). We observed a strong correlation of the first factor (9.218) that falls within the current methodological norms.

In Table 5 we present the matrix of the factorial model that indicates the grouping of the items into a single dominant factor.

### 3.3. Internal Consistency and Reliability

In our study, following the development of the items of the MPIS, we found that this scale has adequate fidelity according to other current methodological norms. The correlation between the items and the total score of the MPIS (between 0.52 and 0.55) is significant at the threshold *p* < 0.01 indicating the usefulness of keeping the items in the MPIS.

The accuracy and precision of the MPIS were estimated using the Cronbach alpha coefficient. For the total score of MPIS, a coefficient of 0.934 (15 items) was obtained, indicating an excellent internal consistency (Table 6).

To establish the reliability of the MPIS, in the first test phase (T1), a sample of 96 individuals was tested, and after four weeks they were retested (T2). The Pearson Correlation indicated a coefficient of 0.942 (*p* < 0.001), which confirms a good test–retest reliability of the MPIS at this experimental stage of development. The obtained results support the discriminative validity of the MPIS.

In Table 7 we note that MPIS is associated with the Vancouver Scars Measurement Scale (VSS). However, the proposed MPIS scale is not associated completely with the pigmented VSS (r = 0. 078; *p* > 0.05) and with the VSS height (r = 0.091; *p* > 0.05).

Since the items on the VSS are virtually uncorrelated, separate associations of MPIS show that this scale is not a mere measurement of negative emotions alone.

We consider that the results presented in the validation study provide evidence regarding the psychosocial or psychometric worthiness of the MPIS.

The MPIS has several limitations, and as with any self-reporting questionnaire, respondents may consciously skew responses to the extent that they are motivated to do so.

In Table 8, we propose an indicative standard for the native population based on the initial group of 103 patients with scars.

## 4. Discussion

For determining compensation in cases of post-traumatic damage, the principal impact is realized in the alteration of the image perceived by the victim, both for themselves and by other related people, and whether or not the aesthetic damage is recoverable [2,3,12,17].

The victims’ relatives claim that patients internalize, withdraw from social life, and sometimes even become deprived of certain ways to spend their free time, their life becoming less pleasant from the perspective of most activities practiced in the past. Damaged moral values also have repercussions on their professional achievements, and the victims may have difficulty in finding a new job according to their qualifications [2,13,14].

Disfigurement represents a deforming or mutilating bodily injury which is irreversible and may be located anywhere on the body. It can be established only after the depletion of all means offered by plastic and reconstructive surgery. This affects the patients’ appearance, negatively influencing their mental health and well-being, and their adherence to social life, both familial and professional [2,21,22]. The victims are unable to practice a favorite sport/hobby because of the constant awareness of the presence of the scar as unsightly.

Aesthetic damage can be corrected by special medical procedures and only after their depletion can a comprehensible assessment of the possible mutilation be performed. Gender and age are predictors that have negative non-standardized correlation coefficients, and implicitly, an indirect relationship with the criterion hiding scars on the face, while the perception of social support and the foldability of scars have positive correlation coefficients and of course a direct relationship with the criterion hiding face scars. This depends however on the person’s initial appearance, age, and gender [4,23,24]. Usually, a person from an urban area, for example, a young woman, is more affected by a scar than an old man from a rural area.

Scar evaluation procedures should be non-invasive, accurate, reproducible, and easy to use to facilitate objective data collection and clinical utility. Existing methods evaluate parameters such as flexibility, firmness, color, perfusion, thickness, and three-dimensional topography [7,11,19,25]. An improvement of the procedures for objectifying the aesthetic damage is strongly needed to facilitate the victim’s access to compensations likely to cover the repairing of the produced damage. Also, a possible reconciliation or agreement with the accused party would be possible in a shorter time, given the implementation of an objective, measurable method of determining the aesthetic damage, for example, the MPIS, thus avoiding the solely subjective opinions of forensic experts [12,17,26].

In our study, after elaborating the items of the MPIS scale, we observed that this tool offers adequate fidelity by following methodological rules. The MPIS also appreciates the psychosocial impact of scars on patients’ quality of life in so far that a score below 35 points is the equivalent of the psychosocial internalization of the scars, which we can define as adapting and living with a scar, while those over 55 after the depletion of all therapeutical possibilities means disfigurement. The correlation between the items and the total score of MPIS (between 0.52 and 0.55) is statistically significant (*p* < 0.01), indicating the usefulness of these items for the assessment of aesthetic prejudice. The fidelity of the MPIS scale was estimated by employing Cronbach’s alpha coefficient. In the case of the total MPIS score, a coefficient of 0.934 was obtained (15 items). This value indicates a good internal consistency, as can be observed in Table 6.

Remarkably, people with scars have a distorted perception (compared to controls) of the support provided by significant people [t (202) = 2.473; *p* = 0.01]. The total score of MPIS detects similarities between the two groups of participants [t (203) = 0.799; *p* = 0.006], supporting the arguments presented above. Hypothetically, people with scars will most likely exhibit a nuanced socio-cognitive and emotional vulnerability that may be factored in future socio-occupational dysfunctions. Several studies mention that through “significant others,” they want to capture the way they perceive people who are useful to them and to whom they relate in various life contexts such as doctors, psychologists, priests, etc. [15,24,25,27,28].

The obtained data support the discriminative validity of the MPIS as presented in Table 6, where we noted that it is associated with the VSS scale. However, the proposed MPIS scale is not associated with pigmented VSS (r = 0.078; *p* > 0.05) and height VSS (r = 0.09; *p* > 0.05). Given that the items of the VSS scale are virtually uncorrelated, the separate associations of MPIS show that this instrument is not a simple measurement of only negative emotions. We consider that the results presented in the validation study bring evidence regarding the psychometric solidity of the MPIS scale [17,27,28]. We developed this scale for the evaluation of victims of accidents and violence but similarly to the Vancouver scale, which was first developed to assess burn scars, MPIS could be adapted to assess the psycho-social impact of other types of scars, such as those following surgery or oncologic procedures.

The MPIS can be used as a research tool for the assessment of aesthetic injury and by forensic departments and lawyers. This scale has been designed as a clinical tool for the forensic pathologist or medical examiner and provides an easy classification of the impact of a scar, an aesthetic injury on a person’s image of themselves, to more accurately describe the influence it will have on that person’s wellbeing. It can be used as a criterion included in the Penal Code for different categories of crimes, to guide patients towards cosmetic and reconstructive surgery, and last but not least, as a tool for insurance companies to determine the amount of compensation granted to victims.

Important aspects still need to be nuanced, so we consider it necessary to implement studies that comprise additional recommendations. In addition, we tried to cover these issues by employing the MPIS scale both in individuals with scars as well as in controls.

Study limitations: our study has several limitations, the most important being the small size of our study group and the fact that the scale was applied on a heterogeneous population (differences in age, gender, education, ethnicity, religion, and cultural background) not large enough to be analyzed distinctly. The separate analysis of numerous individuals from these subcategories would probably have resulted in supplementary conclusions. Another limitation is that the study was conducted only in two geographical regions of our country, both characterized by a mixture of populations.

## 5. Conclusions

The Mekereș Psychosocial Internalization Scale can play a role in avoiding the subjectivism of the forensic examiner regarding aesthetic injury and proposes clearer limits for its presence or absence. It can be used to supplement the purely morphometric scales employed for the assessment of scars to highlight their impact on the mental and social well-being of affected individuals

## Figures and Tables

**Table 1:** Demographic data for the group of 103 patients with scars and of the control group (*n* = 101).

Variable		Patients with Scars	Control Group	*p*
Gender	Men	51 (49.5%)	47 (46.5%)	0.670
	Women	52 (50.5%)	54 (53.5%)	
Marital Status	Unmarried	31 (30.1%)	27 (26.7%)	0.179
	Married	35 (34.0%)	49 (48.5%)	
	Divorced	15 (14.6%)	13 (12.9%)	
	Widower	17 (16.5%)	12 (11.9%)	
	Stable relationship	5 (4.9%)	0	
Geographical Distribution	Rural	53 (51.5%)	45 (44.6%)	0.323
	Urban	50 (48.5%)	56 (55.4%)	
Ethnicity	Romanian	70 (68%)	73 (72.3%)	0.001 *
	Hungarian	22 (21.4%)	28 (27.7%)	
	Slovak	11 (10.7%)	0	
Education	High school or lower	31 (30.1%)	17 (16.8%)	˂0.001 *
	College	40 (38.8%)	24 (23.8%)	
	Higher education	32 (31.01%)	60 (59.4%)	
Economic level	Low	42 (40.8%)	29 (28.7%)	˂0.001 *
	Medium	38 (36.9%)	64 (63.4%)	
	High	23 (22.3%)	8 (7.9%)	

* Chi-square test or Freeman–Halton extension of Fisher’s exact test, *p* ˂ 0.05.

**Table 2:** Items, mean and standard deviations of the MPIS.

No.	Items		Patients with Scars (*n* = 103)	Men (*n* = 51)	Women (*n* = 52)
1	How attractive did you consider yourself before the occurrence of the scar?	M	3.3592	3.1538	3.5686
		SD	1.02760	0.91576	1.10009
2	How attractive do you feel after the emergence of the scar?	M	2.3495	2.5577	2.1373
		SD	1.09999	1.09210	1.07740
3	Are you aware of the presence of the scar?	M	2.9029	2.4615	3.3529
		SD	1.52441	1.29041	1.62263
4	How much has the presence of the scar changed your life?	M	2.2039	1.6731	2.7451
		SD	1.45763	1.02366	1.63515
5	Was your relationship with other people negatively affected by the presence of the scar/has it influenced your relationship with others?	M	2.1456	1.5385	2.7647
		SD	1.49120	0.97943	1.66839
6	Did you need psychological/psychiatric help before the occurrence of the scar?	M	1.2233	1.1731	1.2745
		SD	0.87371	0.78519	0.96080
7	Did you need psychological/psychiatric help after the emergence of the scar?	M	1.9029	1.3654	2.4510
		SD	1.24077	0.81719	1.36108
8	Have you ever felt anxious before the scar occurred?	M	1.2427	1.1538	1.3333
		SD	0.87968	0.69690	1.03280
9	Did you ever feel anxious after the scar was produced?	M	2.3107	1.5192	3.1176
		SD	1.59059	1.03829	1.65707
10	How has the scar changed your way of interacting with others?	M	2.0971	1.4423	2.7647
		SD	1.47870	0.93753	1.63203
11	Does the presence of the scar have an impact on your sexual behavior?	M	2.0583	1.4423	2.6863
		SD	1.48737	.91638	1.69104
12	Do you think you have a lesser chance of a social or close relationship?	M	2.0680	1.3462	2.8039
		SD	1.52921	0.88306	1.69729
13	Do you think the presence of the scar reduces your chances of getting/keeping a job?	M	2.2913	1.5385	3.0588
		SD	1.57574	1.03775	1.66627
14	When you see the scar, do you remember the former traumatic event accurately?	M	3.4078	2.7885	4.0392
		SD	1.34629	1.28851	1.09473
15	Do you ever try to hide the scar on your face?	M	2.2233	1.4038	3.0588
		SD	1.67409	1.14206	1.72525

Note: M = average; SD = standard deviation.

**Table 3:** Baseline indicators of exploratory factor analysis of the MPIS.

KMO and Bartlett Test ^a^		
Kaiser–Meyer–Olkin for Measuring the Suitability of the Group		0.846
Bartlett sphericity test	χ^2^	2002.302
	Df	105
	*p*	0.000

Note: ^a^ experimental group.

**Table 4:** The factors of the composition of the MPIS and the explained variance.

Component	Initial Eigenvalues			Varimax Rotation
	Total	% from Variance	% Cumulative	Total
Item 1	9.218	61.451	61.451	9.218
Item 2	1.775	11.836	73.288	1.011
Item 3	1.284	8.559	81.846	1.002
Item 4	0.672	4.482	86.329	
Item 5	0.456	3.042	89.371	
Item 6	0.411	2.740	92.111	
Item 7	0.346	2.305	94.416	
Item 8	0.253	1.686	96.101	
Item 9	0.203	1.356	97.457	
Item 10	0.148	0.984	98.441	
Item 11	0.083	0.554	98.996	
Item 12	0.059	0.390	99.386	
Item 13	0.040	0.269	99.655	
Item 14	0.028	0.188	99.843	
Item 15	0.023	0.157	100.000	

**Table 5:** Matrix of the factorial model at the MPIS.

Component	The Matrix of the Factorial Model	Communality
Item 1	0.654	0.827
Item 2	0.797	0.867
Item 3	0.814	0.688
Item 4	0.915	0.839
Item 5	0.924	0.870
Item 6	0.305	0.938
Item 7	0.809	0.726
Item 8	0.766	0.914
Item 9	0.838	0.772
Item 10	0.965	0.936
Item 11	0.944	0.896
Item 12	0.950	0.916
Item 13	0.896	0.806
Item 14	0.777	0.624
Item 15	0.796	0.658

**Table 6:** Fidelity of the Mekereș Psychosocial Internalization Scale.

Scale	Internal Consistency					
	M	AS	Min.	Max.	α Cronbach	α Cronbach Based on Item Standardization
Mekereș Psychosocial Internalization Scale	33.78	15.39	15	72	0.943	0.934

Note: significant at *p* < 0.01.

**Table 7:** The inter-correlation matrix of MPIS with VSS.

	SIPM	VSS Vascularization	VSS Pigmentation	VSS Pliability	VSS Elevation
SIPM	1	0.196 *	0.078	0.279 **	0.091
VSS vascularization	0.196 *	1	0.289 **	0.470 **	0.387 **
VSS pigmentation	0.078	0.289 **	1	0.598 **	0.267 **
VSS pliability	0.279 **	0.470 **	0.598 **	1	0.531 **
VSS elevation	0.091	0.387 **	0.267 **	0.531 **	1

Note: ** significant at *p* < 0.01; * significant at *p* < 0.05; MPIS—Mekereș Psychosocial Internalization Scale; VSS—Vancouver Scar Scale.

**Table 8:** The indicative standard for MPIS.

Media			33.78
Standard Deviation			15.39
The Minimum Value			15
The Maximum Value			72
Percentile		25	15–21
		50	22–28
		75	29–72

## Data Availability

Our data are available on doi:10.17632/xjzrzkgkb/b.1, link: https://doi.org/10.17632/xjzrzkgkbb.1 published online on 3 September 2021.
